# Lower 5-year cup re-revision rate for dual mobility cups compared with unipolar cups: report of 15,922 cup revision cases in the Dutch Arthroplasty Register (2007–2016)

**DOI:** 10.1080/17453674.2019.1617560

**Published:** 2019-05-17

**Authors:** Esther M Bloemheuvel, Liza N van Steenbergen, Bart A Swierstra

**Affiliations:** a Department of Orthopaedic Surgery, Sint Maartenskliniek, Nijmegen;; b Dutch Arthroplasty Register (LROI), ’s Hertogenbosch, the Netherlands

## Abstract

Background and purpose — During revision hip arthroplasty the dual mobility cup (DMC) is widely used to prevent dislocation despite limited knowledge of implant longevity. We determined the 5-year cup re-revision rates of DMC compared with unipolar cups (UC) following cup revisions in the Netherlands.

Patients and methods — 17,870 cup revisions (index cup revision) were registered in the Dutch Arthroplasty Register during 2007–2016. Due to missing data 1,948 revisions were excluded and the remaining 15,922 were divided into 2 groups: DMC (n = 4,637) and UC (n = 11,285). Crude competing risk and multivariable Cox regression analysis were performed with cup re-revision for any reason as endpoint. Adjustments were made for known patient characteristics.

Results — The use of DMC (in index cup revisions) increased from 23% (373/1,606) in 2010 to 47% (791/1,685) in 2016. Patients in the index DMC cup revision group generally had a higher ASA score and the cups were mainly cemented (89%). The main indication for index cup revision was loosening. In the DMC group dislocation was the 2nd main indication for revision. Overall 5-year cup re-revision rate was 3.5% (95% CI 3.0–4.2) for DMC and 6.7% (CI 6.3–7.2) for UC. Cup re-revision for dislocation was more frequent in the UC group compared with the DMC group (32% [261/814] versus 18% [28/152]). Stratified analyses for cup fixation showed a higher cup re-revision rate for UC in both the cemented and uncemented group. Multivariable regression analyses showed a lower risk for cup re-revision for DMC compared with UC (HR 0.5 [CI 0.4–0.6]).

Interpretation — The use of DMC in cup revisions increased over time with differences in patient characteristics. The 5-year cup re-revision rates for DMC were statistically significantly lower than for UC.

Instability and dislocation after total hip arthroplasty (THA) is a common reason for revision surgery according to the implant registers of the Netherlands (22%) and Australia (23%) (LROI 2018, AOANJRR 2017).

The dual mobility cup (DMC) is a “cup in a cup” and was developed in the 1970s to combine the low-friction arthroplasty principle of Charnley with the advantage of a big femoral head principle of McKee to increase implant stability (Philippot et al. 2009). Second, the aim of this product was to decrease polyethylene rim damage from contact between femoral neck and acetabular liner and to restore near-normal range of motion.

Nowadays, the DMC is a well-accepted treatment option for patients with an increased risk for instability in primary and secondary THA (De Martino et al. 2014). However, most literature has focused on dislocation rates rather than on longevity of the implant.

In the Dutch Arthroplasty Register we found a 5-year cup revision rate for DMC of 1.5% (95% CI 1.0–2.3) after primary THA (Bloemheuvel et al. 2019). In the Swedish arthroplasty register Hailer et al. (2012) found a 2-year overall survival percentage of 93% (CI 90–97) for DMC after revision THA.

We studied the cup re-revision rates of DMC using data from the Dutch Arthroplasty Register (LROI) and compared these results with unipolar cup (UC).

## Patients and methods

The Dutch Arthroplasty Register (LROI) started in 2007 and has a completeness of 98% for hip revision arthroplasty (www.lroi-report.nl). The LROI database contains patient, procedure, and prosthesis characteristics. For each component a product number is registered to identify the characteristics of the prosthesis, such as dual mobility or conventional cup.

The vital status of all patients is obtained on a regular basis from Vektis, the national insurance database on health care in the Netherlands, which records all deaths of Dutch citizens.

**Figure 1.**
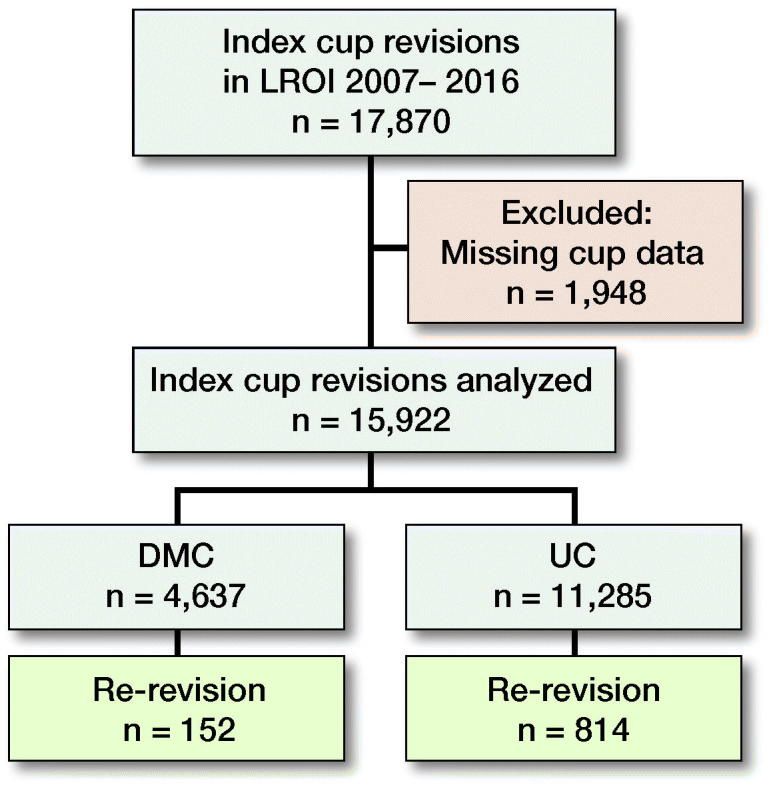
Patient flow. DMC: dual mobility cup; UC: unipolar cup.

For this study we included all index cup revisions in the period 2007–2016. An index cup revision was defined as the 1st registered cup revision, isolated or as part of a total hip revision. A cup re-revision was defined as a procedure where at least the cup was exchanged or removed. Closed reduction after a dislocation or incision and drainage for infection without component exchange were not included in the LROI. Information from the primary (index) procedure is only known when the procedure was performed after 2007 and registered in the LROI. Records with a missing cup product number (n = 1,948) were excluded from the 17,870 index cup revisions registered. Thus, 15,922 index cup revisions were analyzed and divided into DMC (n = 4,637) or UC (n = 11,285) (Figure 1). The median follow-up was 6 (2–11) years.

### Statistics

The index UC and DMC revisions were described separately concerning patient and procedure characteristics. Survival time was calculated as the time from index cup revision to cup re-revision for any reason, death of the patient, or end of the follow-up (January 1, 2018). Cumulative crude incidence of cup re-revision was calculated using competing risk analysis, where death was considered to be a competing risk (Lacny 2015). In addition, Kaplan–Meier survival analyses were performed.

Multivariable Cox proportional hazard analyses were performed to compare DMC and UC. Adjustments were made for sex, age at surgery, ASA score, and type of fixation to discriminate independent risk factors. BMI, Charnley score, and smoking status were not included as covariates, as these were only available in the LROI database since 2014.

For all covariates added to the model, the proportional hazards assumption was checked by inspecting log-minus-log curves and met.

Reasons for cup re-revision were described and compared using a chi-square test. P-values below 0.05 were considered statistically significant. For the 95% confidence intervals (CI), we assumed that the number of observed cases followed a Poisson distribution.

### Ethics, funding, and potential conflicts of interests

The dataset was processed in compliance with the regulations of the LROI governing research on registry data. No external funding was received. No competing interests were declared.

## Results

**Figure 2.**
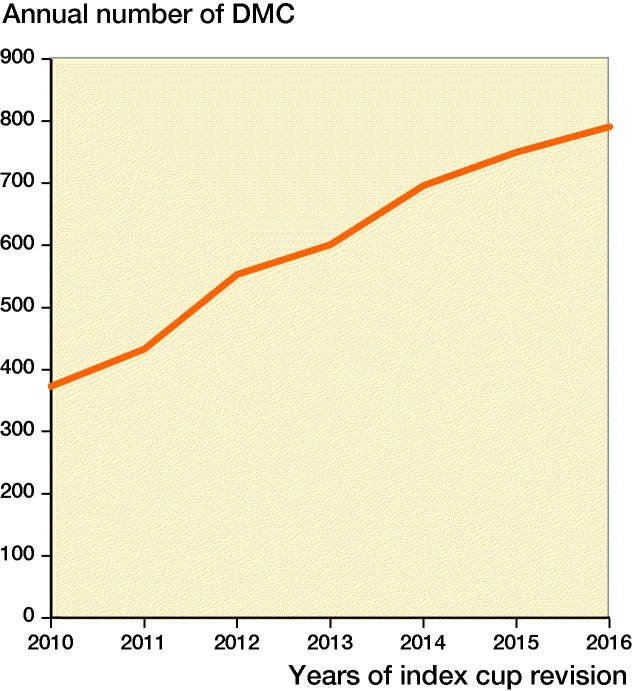
Trend in use of the dual mobility cup (DMC) in revision hip arthroplasty in the period 2010–2016 in the Netherlands (n = 4,637).

The use of DMC (in index cup revisions) increased from 23% (373/1,606) in 2010 to 47% (791/1,685) in 2016 (Figure 2) with 8 different types of DMC used (Table 1).

**Table 1.** Types of dual mobility cups used in index cup revision in the period 2007–2016 in the Netherlands (n = 4,637)

Type	Cemented	Cementless
Biomet Avantage	3,492	86
Biomet Avantage Reload		167
Biomet Avantage Rev HA		19
Smith & Nephew Polarcup	211	194
Amplitude Saturne	250	43
Mathys SeleXys DS Cup	106	35
Groupe LEpine Cupule Quattro	32	
Groupe Lepine Cupule HAP Press-F		2

Patients who received a DMC had a higher ASA score and 89% of the DMC group was cemented versus 59% of the UC group. The most frequent indication for index cup revision was loosening of the acetabular component (37–47%) in both groups. Dislocation was more frequently registered as reason for revision in DMC (35% vs. 12%), while (suspicion of) infection was more frequently registered in the UC group (15% vs. 4%) (Table 2).

**Table 2.** Patient characteristics in index cup revisions according to type of cup. Values are frequency (%) unless otherwise specified

	DMC	UC
Factor	n = 4,637	n = 11,285
Male sex	1,445 (31)	3,692 (33)
Age, mean (SD)	74 (10)	71 (12)
ASA, n (%)		
I	478 (11)	1,824 (18)
II	2,642 (60)	6,307 (60)
III–IV	1,287 (29)	2,308 (22)
Fixation cup		
Cemented	4,057 (89)	6,468 (59)
Uncemented	487 (11)	4,554 (41)
Type of revision		
Partial (cup only)	3,203 (69)	6,411 (57)
Total revision	1,434 (31)	4,874 (43)
Reason for index revision **^a^**		
Loosening acetabular component	1,728 (37)	5,320 (47)
Dislocation	1,619 (35)	1,301 (12)
Infection	185 (4)	634 (15)
Loosening femoral component	673 (15)	2,120 (19)
Girdlestone/spacer	167 (4)	534 (5)
Periprosthetic fracture	223 (5)	445 (4)
Cup/liner wear	665 (14)	1,278 (11)
Peri-articular ossification	157 (3)	387 (3)
Symptomatic metal-on-metal bearing	234 (5)	818 (7)
Other	707 (15)	2,592 (23)

Numbers do not add up to total due to missing data.

DMC: dual mobility cup; UC: unipolar cup.

a The total proportion is over 100% since more than 1 reason for revision can be registered.

Over half of the cup re-revisions were performed for loosening of the acetabular component. Dislocation was the 2nd most frequent reason for cup re-revision (32%) in the UC group, while this was 18% in the DMC group. Suspicion for infection was the 2nd most frequently registered reason (32%) for cup re-revision in the DMC group, compared with 16% in the UC group (Table 3). From the 79 DMC cup re-revisions that loosened, 67 were cemented.

**Table 3.** Reason for cup re-revision according to type of acetabular cup. Values are frequency (%)

Reason for re-revision **^a^**	DMC n = 152	UC n = 814
Loosening acetabular component	79 (52)	423 (52)
Dislocation	28 (18)	261 (32)
Infection	48 (32)	127 (16)
Loosening femoral component	12 (8)	61 (8)
Girdlestone/spacer	20 (12)	44 (5)
Periprosthetic fracture	8 (5)	43 (5)
Cup/liner wear	7 (5)	27 (3)
Peri-articular ossification	2 (1)	17 (2)
Symptomatic metal-on-metal bearing	2 (1)	11 (1)
Other	15 (10)	88 (11)

a See Footnote under Table 2

**Figure 3.**
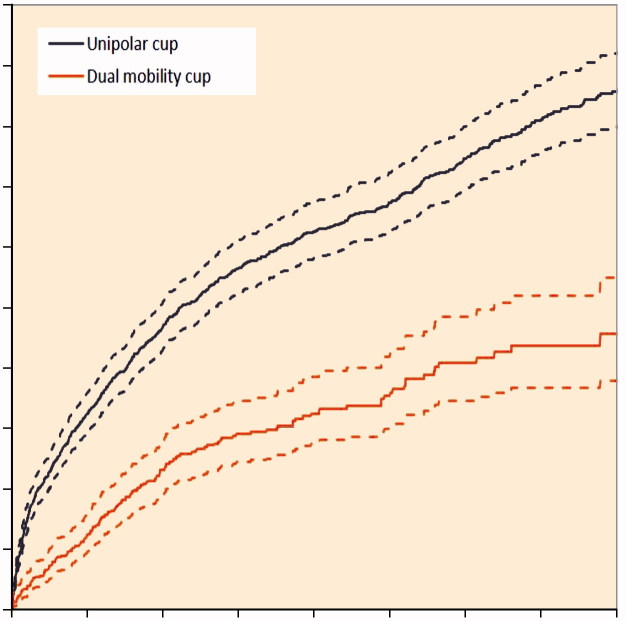
Cumulative incidence of cup re-revision according to type of cup in the period 2007–2016 in the Netherlands (n = 15,922).

The 5-year crude re-revision rate of DMC was 3.5%(CI 3.0–4.2) and 6.7% (CI 6.3–7.2) for UC (Figure 3). Stratified analyses according to type of cup fixation (cemented versus uncemented) showed comparable differences in 5-year crude cumulative incidence of re-revision in favor of the DMC group, both using competing risk analysis (Table 4) and Kaplan-Meier survival analysis (Table 5).

**Table 4.** Crude 5-year cumulative incidence (%) of cup re-revision according to type of acetabular cup. Competing risk was used

	5-year cumulative incidence of cup-re-revision			
	Dual mobility cup		Unipolar cup	
Factor	n	% (CI)	n	% (CI)
Overall	4,637	3.5 (3.0–4.2)	11,285	6.7 (6.3–7.2)
Cup fixation				
Cemented	4,057	3.6 (3.0–4.4)	6,466	7.4 (6.7–8.1)
Uncemented	487	3.7 (2.3–6.0)	4,554	5.7 (5.1–6.5)

**Table 5.** 5-year cumulative incidence (%) of cup re-revision according to type of acetabular cup using Kaplan–Meier survival analyses

	5-year cumulative incidence of cup-re-revision			
	Dual mobility cup		Unipolar cup	
Factor	n	% (CI)	n	% (CI)
Overall	4,637	3.8 (3.2–4.4)	11,285	6.9 (6.3–7.5)
Cup fixation				
Cemented	4,057	3.9 (3.1–4.6)	6,466	7.7 (6.9–8.5)
Uncemented	487	3.8 (3.6–4.0)	4,554	5.9 (5.1–6.7)

Multivariable survival analyses showed an adjusted hazard ratio of 0.5 (0.4–0.6) for re-revision of DMC compared with UC. Adjustments were made for sex, age at surgery, ASA score, and type of fixation to discriminate independent risk factors.

## Discussion

This large register study in the Netherlands showed lower cup re-revision rates of DMC compared with UC.

Currently, DMC is increasingly used in both primary and revision hip arthroplasty (Darrith et al. 2018, Bloemheuvel et al. 2019). A recent systematic review from Darrith et al. (2018) containing all English-language articles dealing with dual mobility (primary and revision) arthroplasty between 2007 and 2016 showed low rates of dislocation (primary 0.5% and revision 2%). The overall survival of the DMC in revision THA was 97% at a mean of 5 years. A limitation of this study is that it could not distinguish between total and partial revisions.

The number of register studies of revision DMC is scarce. Gonzalez et al. (2017) compared DMC and UC THA for prevention of dislocation after revision THA. In this prospective hospital registry-based cohort including all total and cup-only revision THAs (n = 316) they found a lower incidence of dislocation in the case of a DMC (2.7% versus 7.8%) but did not study the longevity of the implant.

In 2012 a register study based on 228 patients from the Swedish Hip Arthroplasty Register showed 7% overall re-revision rates for any reason after a DMC at 2 years follow-up (Hailer et al. 2012). Until our study, this was the only register study focusing on re-revision rates according to type of cup. We cannot compare their outcome with our results, as our endpoint was cup re-revision and not overall re-revision.

A limitation of register studies is the risk for selection bias. It is possible that different cup designs were used for different types of revisions or different types of patients. Therefore, we examined the patient characteristics in detail. We found higher ASA scores in the DMC group, but after correction for casemix factors DMC still showed lower 5-year revision rates compared with UC. Recent annual reports from the Swedish and Australian hip registers found higher ASA scores in case of revision surgery. (AOANJRR 2016, SHAR 2016). However, they did not distinguish between types of cup.

Besides differences between patient characteristics we also examined differences in fixation method. In our study 89% of DMC were cemented, compared with 59% in UC. The amount of cup re-revision because of loosening was the same in the DMC and UC group (52%). We performed stratified analyses to correct for difference in fixation method between DMC and UC and still found a lower cup re-revision rate for DMC compared with UC. The annual report from Sweden showed a trend towards an increased use of cemented DMC in cup revisions (34% of the revision cases received a cemented Avantage cup) (SHAR 2016). However, these revision data were not analyzed in subgroups, for example type of fixation.

It is also interesting to analyze differences between various DMC designs as the choice of implant might depend on doctor or hospital preferences. Hopefully, after a few more years the numbers will increase and we shall be able to do further analyses. Nevertheless, register studies have a limited possibility to analyze differences in patient characteristics as this depends strongly on the number of registered variables. Therefore, registries should be taken along with prospective cohort studies, in order to collect a more extensive set of patient variables.

Our database on revision hip arthroplasties does not contain information on the procedures performed before the start of the LROI in 2007. Therefore, we do not know the type and follow-up of the primary procedure as well as the primary diagnosis of the patient. We do not know whether the 1st revision procedure (defined as index revision) included in our revision hip arthroplasty database was really the 1st revision of a hip or a consecutive revision procedure. On the other hand, including all revision hip arthroplasties available in the LROI resulted in the largest population-based study to date of almost 18,000 cup revisions with a median follow-up of 6 years.

In summary, the use of DMC in cup revisions increased over time with differences in patient characteristics and indications. The 5-year cup re-revision rates for DMC were statistically significantly lower than for UC. This promising mid-term result justifies continued use of DMC in revision hip arthroplasty in anticipation of longer term results.
